# Brief advice and active referral for smoking cessation services among community smokers: a study protocol for randomized controlled trial

**DOI:** 10.1186/s12889-016-3084-z

**Published:** 2016-05-11

**Authors:** Yi Nam Suen, Man Ping Wang, William Ho Cheung Li, Antonio Cho Shing Kwong, Vienna Wai Yin Lai, Sophia Siu Chee Chan, Tai Hing Lam

**Affiliations:** School of Nursing, the University of Hong Kong, 4/F, William MW Mong Block, 21 Sassoon Road, Pokfulam, Hong Kong; Hong Kong Council on Smoking and Health, Unit 44-2-03, 44/F, Hopewell Centre, 183 Queen’s Road East, Wan Chai, Hong Kong; School of Public Health, the University of Hong Kong, 5/F, William MW Mong Block, 21 Sassoon Road, Pokfulam, Hong Kong

**Keywords:** Active referral, Brief intervention, Smoking cessation, Randomized controlled trial

## Abstract

**Background:**

Most smokers do not use smoking cessation (SC) services although it increases successful quits. Passive referral providing SC information to smokers is commonly used in SC studies. Little was known about active referral in the community setting. This study aims to motivate community smokers to quit by brief SC advice using a validated AWARD model (Ask, Warn, Advise, Refer and Do-it-again) that adjunct with active referral of smokers to various SC services in Hong Kong.

**Methods/Design:**

This is a single-blinded, parallel three-armed cluster randomized controlled trial (RCT) with two treatment groups of (1) brief SC advice using the AWARD model, active referral to SC services plus a referral card and a health warning leaflet (active referral group) and (2) brief SC advice using AWARD model and health warning leaflet (brief advice group) and a control group receives general very brief advice with a self-help booklet. A total of 1291 smokers will be recruited from 66 clusters (recruitment sessions) with 22 will be allocated to each of the two intervention and one control groups. SC ambassadors will be trained for delivering the interventions and conducting telephone follow-up. The primary outcomes are self-reported 7-days point prevalence (PP) abstinence at 3 and 6 months follow-up. Intention-to-treat principle and multi-level regressions will be used for data analysis.

**Discussion:**

This is the first RCT on assessing a model combining brief advice and active referral to SC services among community smokers. The results will inform the practices of SC services and intervention studies.

**Trial registration:**

NCT02539875 (ClinicalTrials.gov registry; registered retrospectively on 22 July 2015)

## Background

Smoking remains as a leading preventable cause of death and healthcare costs worldwide [1] and in Hong Kong (HK) [2]. Although the daily cigarette smoking prevalence in HK halved from 23.3 % in 1985 to 10.5 % in 2015 [3], smoking and second-hand smoke accounted for 16 % of the overall number of deaths (*n* = 43,397) in 2013 [4]. Smoking cessation (SC) is highly cost-effective when compared to other health interventions [5]. The World Health Organization MPOWER policy package of “offer help to quit” means proactive SC services are needed to encourage smokers to quit smoking [1]. Current clinical SC practice guidelines of 5As *(****A****sk*, ***A****ssess*, ***A****dvise*, ***A****ssist* and ***A****rrange)* also emphasizes assisting smokers to quit [6]. Most of current SC services use the passive method to recruit smokers that rely on smokers’ self-initiation to seek help, such as calling the quit-line or attending the SC clinics [7]. The impact (effect of SC multiple by the number of smokers covered) [8] of the SC services is limited as only 16 % of smokers seek SC services worldwide [1]. It is worth-noting that HK smokers’ awareness of the SC services has been reduced (70.3 % in 2012 vs. 59.1 % in 2015) and hence the prevalence of services usage among these smokers dropped as well (8.5 % vs. 5.0 %) [3]. This suggests SC services promotion may be sufficient but further increase of usage needs innovative intervention. Thus, smokers should be introduced with, motivated to use and be proactively referred to the service to increase its impact.

Referral intervention includes passive and active methods. Passive referral involves asking or encouraging the smokers to use the SC services (e.g. quit-line or clinics) by providing information sheet of the service [9]. Smokers have to contact the service providers by their own effort and often only a small promotion of smokers will do so. In contrast, active referral involves physicians or other healthcare professionals formally referring smokers (sending smokers’ information, mainly the contact method) to SC services via fax, mail or centralized computer system [9–12], which overcomes the barrier of self-initiation. Once the SC service providers receive smokers’ information, they would subsequently call the smokers for arranging further cessation interventions. The effect of active referral for SC may be larger than that of passive referral as most who were passively referred to SC services failed to call the quit-lines for assistance [13, 14]. Particularly, Borland et al. [10] reported that fax referring smokers to an evidence-based quit-line service doubled the quit rate of the standard in-practice general practitioner management at 12-month follow-up (12.3 % vs 6.9 %).

Evidence on the effects of active referral on smoking abstinence and SC services use were scarce [15]. Most of these studies were conducted in the clinical settings, which showed the feasibility of utilising centralized manpower and patients’ database for developing new models of SC services. However, it is less clear about the feasibility and effectiveness of active referral in the community setting, where accounts for the majority of the smokers who mostly do not actively seek SC services. Smokers in the community may be different to those who are attending the clinics regarding levels of addiction, quit attempts and intention [16, 17]. Unlike clinical settings, lengthy interventions are less feasible in the community setting and community smokers have no prior rapport with the interventionists. Thus, a brief on-site intervention model is more feasible and subsequent intensive intervention can be referred to current SC services.

Based on the established SC guidelines [18], we have refined the SC guideline and developed a brief validated SC intervention using AWARD model: *Ask, Warn, Advise, Refer and Do-it-again* for community smokers [19]. Short advice lasting for 30 s to 10 min was found to be feasible in the community setting [16]. While most of the previous studies only focused on the quit-line referral (only one clinic referral study is identified [15]), there is limited knowledge on the effectiveness of referral to various SC services including quit-line, clinics and traditional acupuncture treatments. This RCT aims to motivate smokers to quit using brief intervention (AWARD) and actively refer smokers to major SC services in HK.

## Methods/Design

### Overview of design

**Fig. 1:**
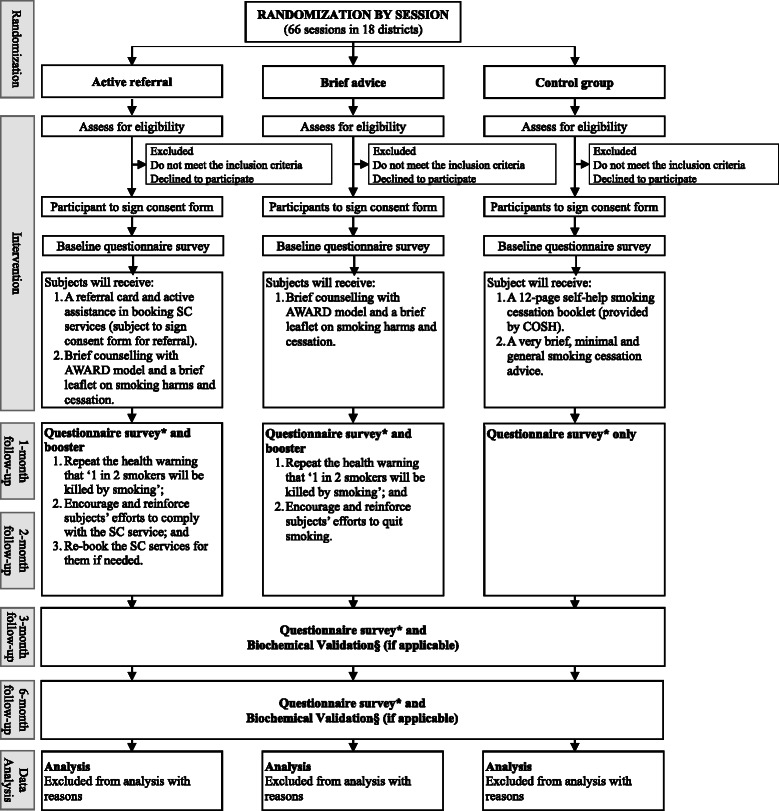
Study design. *Survey includes participants’ demographics (baseline only), smoking habits, past quitting history (baseline only), quitting progress, readiness and efficacy to quit and their knowledge about smoking are obtained at recruitment site as the baseline; and § biochemical validation includes exhaled carbon dioxide and saliva cotinine tests

This is a single-blinded, parallel three-armed cluster RCT with 1291 smokers aged 18 or above will be recruited from the community (Fig. 1). Cluster randomization will be used based on the recruitment sessions (total 66 sessions) to assign participants to one of three conditions of (1) brief SC advice using AWARD model, active referral to current SC services plus a referral card and a health warning leaflet (active referral group); (2) brief SC advice using AWARD model and health warning leaflet (brief advice group); or (3) self-help booklet with general advices (control group). The trial will follow the CONSORT criteria [20].

### Subject inclusion and exclusion criteria

Inclusion criteria include (1) Hong Kong residents aged 18 or above; (2) smoke at least one cigarette per day for the past 3 months; (3) exhaled carbon monoxide (CO) ≥ 4 ppm; (4) intent to quit or reduce smoking [21]; (5) able to communicate in Cantonese (including reading Chinese) and (6) have a local phone number for follow-up. Smokers who meet the above criteria but are currently involved in other SC programs and/or mentally or physically unfit for communication will be excluded.

### Recruitment

Participants will be recruited over 3 months from 66 recruitment sessions in the community in all 18 districts in Hong Kong as in our previous “Quit-to-Win” project [19, 22, 23]. Public open places with high flow of smokers will be identified and these mostly include outdoor places near large shopping malls or housing estates. Booths containing SC and recruitment messages will be set up onsite to attract smokers. Well-trained SC ambassadors will actively recruit the smokers who are near or attending the booths. The SC ambassadors are trained university students (mainly from the fields of nursing, public health, science and social sciences) and volunteers from the non-governmental organizations (NGOs). The SC ambassadors have to attend a two-day workshop in which they learnt the basic knowledge on smoking, tobacco control, SC services, skills on recruitment and intervention delivery. We will adopt “a-foot-in-the-door” approach [24] by asking the smokers their interests on participating in the SC trial. Smokers who showed interests will be assessed for eligibility and informed consents will be sought. The study has been approved by the Institutional Review Board of the University of Hong Kong/Hospital Authority Hong Kong West Cluster (UW15-332) and registered at ClinicalTrials.gov (NCT02539875).

### Cluster randomization

All participants recruited in a particular recruitment activity will be allocated to one of the 3 RCT groups. Individual randomization will not be used as the risk of intervention contamination at each site is high. Cluster randomization will be used to ensure the number of recruitment activities for the three RCT groups is similar. Sixty-six recruitment activities will be randomly assigned to any one of the three RCT groups by randomly generating blocks of 3, 6 or 9. The investigator will generate a list of random numbers in each block using Microsoft Excel. The list of random numbers will be assigned to a list of group assignment in which the first, second and third tertile will be as the active referral, brief advice and control groups respectively. A co-investigator who is not aware of allocation group will subsequently sort the random number in each block and that will be the group assignment sequence of the recruitment activities.

### Blinding

The RCT is single-blinded that all outcome assessors are not aware of the group assignment of the subjects.

### Sample-size calculation

The sample size calculation is based on the primary outcome of self-reported 7-day PP quit rate at the 3-month follow-up. Based on the previous similar studies conducted in the community in Hong Kong, the 3-month quit rate for the control group was approximately 10.0 % [19, 22, 23]. According to the RCT of active referral conducted by Borland et al. [10], the rate ratio of quit rate for the intervention and control group was 1.92 (intervention group: 12.3 %, control group: 6.9 %). Therefore, the effect size for the intervention in this study is set conservatively at 1.60. The quit rate for the intervention group (a combination of active referral and brief advice groups) versus the control group is 16.0 and 10.0 % respectively. To detect a significant difference of quit rate between intervention and control groups with a power of 80 and 5 % significance level, we will need 284 subjects per group. Assuming an intra-cluster correlation coefficient as 0.005 and retention rate of 70 % at the 3-month follow-up, the total sample size taking into account in the intra-cluster correlation within each recruitment session and attrition is 1291.

### Description of the intervention

Each RCT group consists of one or a combination of two/three of the following component as the intervention. The detail is shown in Table 1.

**Table 1:** Summary of intervention in 3 groups

	Active referral group	Brief advice group	Control group
Active referral and referral card	✓		
Brief counselling with AWARD model	✓	✓	
Smoking harms and cessation leaflet	✓	✓	
General Advice and self-help booklet			✓

#### Active referral

Subjects will receive a referral card and be actively referred to one of the five major SC services in Hong Kong, including (1) Department of Health SC Services, (2) Tung Wah Group Hospital Integrated Center on SC, (3) Hospital Authority (HA) SC Clinics, (4) Pok Oi Hospital Chinese Medicine SC Services, and (5) University of Hong Kong Youth Quit-line. The details of each service are shown in Table 2. The well-trained SC ambassadors will explain the SC services to the subjects who will be asked to choose one of the SC services to be referred. Informed written consent and contact telephone number of the subjects will be obtained at the recruitment site, processed primarily in the university research office (including putting subjects’ information into computer), transferred to the collaborator - COSH (Hong Kong Council on Smoking and Health) and eventually destined at the selected service provider within 7 days. As proactive phone call service is not available in HA Clinics, subjects will be encouraged to make the appointment by themselves using a provided list of HA clinics contact addresses and telephone numbers. Research staff will monitor SC services use of the subjects and assist them to make or re-make the appointment if necessary in each follow-up (1-, 2-, 3- and 6- month).

**Table 2:** Major smoking cessation services in Hong Kong

	Main services
Department of Health	Phone SC counselling managed by registered nurses.
Hospital Authority	SC clinics run by physicians and nurses.
	Free nicotine replacement therapy (NRT) and cessation drugs.
Tung Wah Group of Hospitals	SC clinics run by physician, nurses, and social worker.
	Free nicotine replacement therapy (NRT) and cessation drugs.
Pok Oi Hospital	SC clinics run by Chinese medical practitioners.
	Free acupuncture cessation therapy.
Youth Quit-line	Telephone counselling by well-trained youth smoking cessation counsellors.

#### Referral card

The pocket size referral card includes three major parts: (1) an introduction on each of the existing SC services; (2) practical information including the cessation hotline, address, operation hours of the SC clinics and address, and 3D barcodes of online SC services; and (3) highlights of information that may motivate smokers to use the service including assistance provided by experienced professional SC nurses or physicians; various cessation methods such as telephone counselling, face-to-face counselling, nicotine replacement therapy, cessation medication and acupuncture, which suit individual preference and are free of charge, convenient in access, and individual support if needed at follow-ups. The card is served as a reminder of information of SC services available.

#### AWARD model

Subjects will receive brief counseling using AWARD model that is an innovative, simple and effective model to guide the counselling [19]. It is more feasible than more lengthy or intensive counselling and can be delivered by lay or minimally trained person in community settings. AWARD model includes five components and can be delivered within a minute: (1) Ask about smoking history; (2) Warn about the high risk of smoking; (3) Advise to quit as soon as possible and comply with the decided quit date; (4) Refer smokers to smoking cessation services; and (5) Do it again.

#### Health warning and SC information leaflet

A 2-side colour printed A4 leaflet is designed to cover the most important messages to motivate participants to quit smoking. The content of the leaflet includes: (1) highlights of the absolute risk of death due to smoking; (2) the whole list of diseases caused by active and second-hand smoking; (3) ten horrible pictorial warnings of health consequences of smoking and secondhand smoke in one page to maximize the impacts; (4) benefits of smoking cessation and (5) simple messages to encourage participants to quit smoking and remind them to call the Department of Health SC hotline 183 3183.

#### General advice and a self-help smoking cessation booklet

Participants will receive very brief, minimal and general smoking advice and a 12-page self-help smoking cessation booklet developed by the collaborator (COSH).

### Data collection

#### Baseline

Demographics including gender, age, marital status, number of children, type of residence, educational level, employment status and household income will be collected. Average number of cigarettes smoked per day, the age starting smoking and the usual time having the first cigarette smoked each day, attempts to quit or reduce, methods used in past quitting attempts, reasons of not currently using the SC services, readiness (decisional date to quit) and perceived importance, difficulties and confidence to quit smoking, and knowledge about smoking (e-cigarette and risk of smoking) will be collected using validated questions.

#### Follow-up

Follow-up telephone calls will be conducted at 1-, 2-, 3- and 6-months after the baseline to assess changes in smoking habits and progress of quitting especially the use of referred SC services. Subjects who reported not smoking in past 7 days at the 3- and 6-month follow-up will be furthered assessed by inviting their relatives/friends for verification (non-biochemical validation) and will be biochemically validated using the exhaled carbon monoxide and saliva cotinine tests.

### Outcome assessments

#### Primary outcome

The primary outcomes are self-reported 7-days PP quit rate at 3-month and 6-month follow-up. Subjects reporting not smoking in the past 7-days at 3-month and 6-month will be regarded as abstinence from smoking.

#### Secondary outcomes

Secondary outcomes at 3- and 6-month follow-up include:

1. SC service use and indicators of use: calling a hotline, making an SC appointment, SC clinic attendance and counselling session attendance. Information of self-reported SC service use of active referral group will be verified with service providers’ records.
2. Biochemical validated smoking abstinence: Smoking abstinence will be defined as exhaled carbon monoxide (CO) level <4 ppm and saliva cotinine level <10 ng/ml [25, 26].
3. Smoking reduction: cigarette consumption reduced by at least 50 % compared with the baseline.

### Data analysis

The main comparisons will be the self-reported 7 days PP quit rate among groups, which include: (1) Active referral plus brief advice vs. control: to test the bulk model of active referral with referral card plus brief counselling using AWARD model with smoking harms and cessation leaflet intervention effects; (2) Brief advice vs. control: to test brief counselling using AWARD model with smoking harms and cessation leaflet intervention effects; and (3) Active referral vs. brief advice: to test active referral with referral card intervention effects.

The intention-to-treat (ITT) principle will be used for outcome comparison between groups. Information missed at follow-up will be considered as non-quitters, non-reducers or do not use SC service if no records from the service providers are available for verification. Methods to handle missing cases (multiple imputations or complete case analysis) will depend on the proportion of actual percentages of the attrition rate. Baseline characteristics and outcome measures among groups at each endpoint will be compared using chi-square tests, Mann–Whitney tests and *t*-tests. Logistic regression will be used to predict quitting and models will be adjusted for baseline differences if necessary. Multi-level analysis method will be used to handle clustering effects.

## Discussion

To the best of our knowledge, this is the first RCT to test the effectiveness of a model combining brief SC advice and active referral smoking cessation intervention in the community setting. Unlike recruiting smokers in the clinical setting where smokers may be more motivated by the undesirable health condition, recruiting smokers in the community may be more difficult. Brief SC advice and referral provided by physicians and other healthcare professionals in the clinical setting has been found effective [27, 28], our study leads to deliver SC advice and refer smokers to SC services by training the university students and volunteers from the NGOs, which is cheaper than health care professionals. This study is unique in several ways. We will refer smokers not only to the SC quit-lines but also to the SC clinics (Western and Traditional Chinese Medicine) upon smokers’ preference. Such strategy allows smokers to choose a service that they believe to be most useful and hence shall increase their adherence to the service. We will evaluate the effectiveness of the intervention not solely by the abstinence rate, but also assessing smokers’ substantial use of referred service and their comments over the service.

If the intervention is proven feasible and effective in SC, it could ease the burden made by smoking on the medical system as the referral and SC advice are not necessarily to be done by healthcare professionals and hopefully result in less medical cost on diseases that are attributed to smoking. The study will also demonstrate the importance of partnership between SC services on the promotion of SC in the community.

### Ethical approval and consent

This study has received ethical approval by the Institutional Review Board of the University of Hong Kong/ Hospital Authority Hong Kong West Cluster (IRB reference no.: UW15-332). The study poses minimal additional risk to study participants. Informed consent will be obtained from the participants for their participation in the study and agreement to allow us to transfer their contact information to their chosen SC service providers (for Group A only).

## Abbreviations

AWARD: Ask, Warn, Advice, Refer and Do-it-again
CO: carbon monoxide
CONSORT: consolidated standards of reporting trials
COSH: Hong Kong Council on Smoking and Health
HA: Hospital Authority
HK: Hong Kong
ITT: intention-to-treat
NGOs: non-governmental organizations
NRT: nicotine replacement therapy
PP: point prevalence
RCT: randomized controlled trial
SC: smoking cessation

## References

[1] World Health Organization. Tobacco Free Initiative. 2015. http://www.who.int/tobacco/mpower/en/. Accessed 1 May 2015.

[2] Lam TH, Ho SY, Hedley AJ, Mak KH, Peto R (2001). Mortality and smoking in Hong Kong: case–control study of all adult deaths in 1998. BMJ.

[3] Census and Statistics Department (2016). Thematic Household Survey, Report No.59: Pattern of Smoking.

[4] Census and Statistics Department (2014). The Mortality Trend in Hong Kong, 1981 to 2013.

[5] Jha P, Chaloupka FJ (1999). Curbing the epidemic: governments and the economics of tobacco control.

[6] Fiore MC, Jaén CR, Baker TB, et al. Treating Tobacco Use and Dependence: 2008 Update. Clinical Practice Guideline. Rockville, MD: U.S. Department of Health and Human Services. Public Health Service; 2008.

[7] McDonald PW (1999). Population-based recruitment for quit-smoking programs: an analytic review of communication variables. Prev Med.

[8] Glasgow RE, Vogt TM, Boles SM (1999). Evaluating the public health impact of health promotion interventions: the RE-AIM framework. Am J Public Health.

[9] Guy MC, Seltzer RG, Cameron M, Pugmire J, Michael S, Leischow SJ (2012). Relationship between smokers’ modes of entry into quitlines and treatment outcomes. Am J Health Behav.

[10] Borland R, Balmford J, Bishop N (2008). In-practice management versus quitline referral for enhancing smoking cessation in general practice: a cluster randomized trial. Fam Pract.

[11] Vidrine JI, Shete S, Cao Y (2013). Ask-Advise-Connect: a new approach to smoking treatment delivery in health care settings. JAMA Intern Med.

[12] Lewis KE, Durgan L, Edwards VM, Dixon H, Whitehead C, Sykes RN (2009). Can smokers switch from a hospital-based to a community-based stop smoking service? an open-label, randomized trial comparing three referral schemes. Nicotine Tob Res.

[13] Borland R, Segan CJ (2006). The potential of quitlines to increase smoking cessation. Drug Alcohol Rev.

[14] Bentz CJ, Bayley KB, Bonin KE, Fleming L, Hollis JF, McAfee T (2006). The feasibility of connecting physician offices to a state-level tobacco quit line. Am J Prev Med.

[15] Murray RL, Coleman T, Antoniak M (2008). The effect of proactively identifying smokers and offering smoking cessation support in primary care populations: a cluster-randomized trial. Addiction.

[16] Hahn EJ, Rayens MK, Chirila C, Riker CA, Paul TP, Warnick TA (2004). Effectiveness of a quit and win contest with a low-income population. Prev Med.

[17] Lando HA, Pirie PL, McGovern PG, Pechacek TF, Swim J, Loken B (1991). A comparison of self-help approaches to smoking cessation. Addict Behav.

[18] Tobacco Use and Dependence Guideline Panel (2008). Treating tobacco use and dependence: 2008 update.

[19] Chan SS, Wong DC, Cheung YT (2015). A block randomized controlled trial of a brief smoking cessation counselling and advice through short message service on participants who joined the Quit to Win Contest in Hong Kong. Health Educ Res.

[20] Schulz KF, Altman DG, Moher D, Consort Group (2011). CONSORT 2010 statement: updated guidelines for reporting parallel group randomised trials. Int J Surg.

[21] Hyland A, Borland R, Li Q (2006). Individual-level predictors of cessation behaviours among participants in the International Tobacco Control (ITC) Four Country Survey. Tob Control.

[22] Chan SCC, Wong DCN, Cheung DYT (2014). “Quit to Win 2012” and smoking cessation.

[23] Chan SSC, Wong DCN, Lau LMM, Lai VWY, Lam COB, Lam TH (2013). “Quit to Win 2010” and smoking cessation.

[24] Freedman JL, Fraser SC (1966). Compliance without pressure: the foot-in-the-door technique. J Pers Soc Psychol.

[25] Javors MA, Hatch JP, Lamb RJ (2005). Cut-off levels for breath carbon monoxide as a marker for cigarette smoking. Addiction.

[26] Cooke F, Bullen C, Whittaker R, McRobbie H, Chen MH, Walker N (2008). Diagnostic accuracy of NicAlert cotinine test strips in saliva for verifying smoking status. Nicotine Tob Res.

[27] Rice VH, Hartmann-Boyce J, Stead LF (2013). Nursing interventions for smoking cessation. Cochrane Database Syst Rev.

[28] Stead LF, Buitrago D, Preciado N, Sanchez G, Hartmann-Boyce J, Lancaster T (2013). Physician advice for smoking cessation. Cochrane Database Syst Rev.

